# Interplay between Socioeconomic Markers and Polygenic Predisposition on Timing of Dementia Diagnosis

**DOI:** 10.1111/jgs.16406

**Published:** 2020-03-18

**Authors:** Olesya Ajnakina, Dorina Cadar, Andrew Steptoe

**Affiliations:** ^1^ Department of Behavioural Science and Health Institute of Epidemiology and Health Care, University College London London UK; ^2^ Department of Biostatistics & Health Informatics Institute of Psychiatry, Psychology and Neuroscience, Kingʼs College London London UK

**Keywords:** polygenic score, time to dementia diagnosis, genome‐wide association studies, Alzheimerʼs disease, *APOE*‐ε4

## Abstract

**OBJECTIVES:**

Identifying the interplay between socioeconomic markers (education and financial resources) and polygenetic predisposition influencing the time of dementia and the diagnosis of clinical Alzheimerʼs disease (AD) dementia is of central relevance for preventive strategies.

**DESIGN:**

Prospective cohort design.

**SETTING:**

The English Longitudinal Study of Aging is a household survey data set of a representative sample.

**PARTICIPANTS:**

A total of 7,039 individuals aged 50 years and older participated in the study. Of these, 320 (4.6%) were diagnosed with dementia over the 10‐year follow‐up.

**MEASUREMENTS:**

Polygenic score (PGS) for Alzheimerʼs disease (AD‐PGS) was calculated using summary statistics from the International Genomics of Alzheimerʼs Project. An accelerated failure time survival model was used to investigate interactions between AD‐PGS and socioeconomic markers on the timing of dementia and clinical AD dementia diagnosis.

**RESULTS:**

A one standard deviation increase in AD‐PGS was associated with an accelerated time to dementia diagnosis by 4.8 months. The presence of the apolipoprotein E gene (*APOE*‐ε4) was associated with an earlier dementia onset by approximately 24.9 months, whereas intermediate and low levels of wealth were associated with an accelerated time to dementia diagnosis by 12.0 months and 18.7 months, respectively. A multiplicative interaction between AD‐PGS and years of completed schooling in decelerating the time to clinical AD dementia by 3.0 months suggests educational attainment may serve as a protective mechanism against AD diagnosis among older people with a higher polygenic risk. Interaction between AD‐PGS and lower wealth accelerated the time to clinical AD dementia diagnosis by 21.1 to 24.1 months.

**CONCLUSION:**

Socioeconomic markers influence the time to dementia and clinical AD dementia diagnosis, particularly in those with a higher polygenic predisposition. J Am Geriatr Soc 68:1529‐1536, 2020.

As average life expectancy increases, it is projected that the number of people with dementia in England and Wales will increase by 57% by 2040.^1^ In the absence of effective treatments,^2^ delaying dementia diagnosis would confer a great beneficial effect at both individual and societal levels.^3^

Although the causes of dementia are likely to be multifactorial,^4^ robust measures of socioeconomic resources,^5^ such as educational attainment and wealth, are important determinants of dementia diagnosis among older adults.^6^, ^7^ Educational attainment, a status indicator achieved during the first decades of life, has been linked to levels of wealth accumulated in later life, although these two socioeconomic factors characterize different life stages^8^ and could have different pathways influencing the time of dementia diagnosis. There is also a substantial genetic contribution to dementia. Two ε4 alleles of the apolipoprotein E gene (*APOE*‐ε4) are major risk factors^9^ although its presence is neither necessary nor sufficient to develop dementia.^10^

A polygenic score (PGS) provides a unique approach to capturing the cumulative genetic contribution to a condition by combining numerous genetic variants of small effects.^11^ PGSs were proven to have a relatively strong predictive utility for Alzheimerʼs disease (AD) risk.^12^, ^13^, ^14^ However, the role of PGSs in the timing of dementia diagnosis has not been tested. It is also imperative to understand how genetic risk may interact with socioeconomic factors in influencing the time to dementia diagnosis. A higher genetic risk may exacerbate the effect of lower educational attainment and wealth or vice versa. Alternatively, the genetic risk may be independent of socioeconomic characteristics. A clearer understanding of gene‐by‐environment interaction will help highlight potential pathways through which dementia may develop.

We used a large population‐representative cohort of older adults to investigate whether higher PGS calculated from common genetic variants associated with AD was associated with the time to dementia diagnosis. We further tested interactions between these PGSs with educational attainment and wealth in relation to the timing of dementia diagnosis. We hypothesized that older adults with higher PGS would be at greater risk of more accelerated time to a dementia diagnosis. We further hypothesized there would be significant interaction effects between PGS and socioeconomic factors in association with the time to dementia.

## METHODS

### Sample

Data came from the English Longitudinal Study of Aging (ELSA), a nationally representative longitudinal panel study of English adults aged 50 years and older.^15^ The ELSA study began in 2002‐2003 (wave 1) with participants recruited from an annual cross‐sectional survey of households who were then followed up every 2 years, providing detailed information on health and socioeconomic circumstances for each ELSA participant.^15^ The baseline was wave 2 (2004‐2005) for the core members who started at wave 1 or wave 4 for the participants joining the study through the refreshment sample. The genetic data were collected at waves 2 and 4. Follow‐up data were from wave 8 (2016‐2017). We included ELSA participants who were free of dementia at baseline. Ethical approval for each ELSA wave was granted by the National Research Ethics Service (London Multicentre Research Ethics Committee). All participants gave informed consent.

### Study Variables

#### *Ascertainment of Dementia Cases*

To ascertain dementia cases, we used methods with demonstrated utility in population‐based cohorts.^16^, ^17^, ^18^ Dementia diagnosis was ascertained at each wave using self‐report participantsʼ physician diagnosis of dementia or clinical AD dementia. For those ELSA participants who were unable to respond to the main interview themselves, the Informant Questionnaire on Cognitive Decline in the Elderly (IQCODE) was administered with a score above the threshold of 3.38 indicating the presence of dementia.^19^ The selected threshold demonstrated both excellent specificity and sensitivity for detection of all‐cause (undifferentiated) dementia.^20^ Overall, 83.5% of dementia cases were identified from reports of physician‐diagnosed dementia or clinical AD dementia, and 16.5% were identified based on the IQCODE score.

#### *Survival Time*

Time to dementia diagnosis was defined as the period from the baseline when all participants were dementia free to the date when an ELSA participant received the first self‐report physician diagnosis of dementia or the first time of confirmed dementia through the IQCODE assessment during follow‐up. For those without dementia, the survival time was calculated using the period spanning from study entry until the point of their death, or the last wave before dropout, or wave 8. To ascertain the point of death, mortality data were used from the National Health Service central register; all individuals included in the study provided written consent for the linkage.

#### *Socioeconomic Indicators*

Educational attainment at baseline was measured as the number of years of completed schooling. To reflect the accumulation of resources at older ages, wealth was measured at baseline by summing wealth from property, possessions, housing, investments, savings, artwork, and jewelry, and net of debt.^15^ Because incomes among older people often do not reflect the available financial resources very well, it was not included as part of the wealth definition.^15^ To provide more insight into the effects of different levels of wealth, this variable was divided into high, intermediate, and low levels using the interquartile range.

#### *Covariates*

Based on the previous findings,^6^ baseline age, sex, marital status, current smoking status, genetic ancestry (measured with principal components^21^), and *APOE*‐ε4 status were included as covariates. Similar to previous research,^22^ *APOE*‐ε4 status was defined according to the absence (*APOE* ε2/2, ε2/3, and ε3/3) or presence (*APOE* ε2/4, ε3/4, and ε4/4) of *APOE*‐ε4 alleles. Male sex, not married, currently smoking, and the absence of *APOE*‐ε4 alleles were used as reference groups.

### Genetic Data

#### *Quality Control*

Genome‐wide genotyping was performed at University College London Genomics in 2013‐2014 using the Illumina HumanOmni2.5 BeadChips (HumanOmni2.5‐4v1, HumanOmni2.5‐8v1.3). Quality control is described in the Supplementary Tables. Briefly, samples were removed based on call rate (<.99), suspected non‐European ancestry as identified though principal components analysis (see later), and self‐identification, heterozygosity, and relatedness. Single nucleotide polymorphisms (SNPs) were excluded if they were non‐autosomal; the minor allele frequency was less than .01%, if more than 2% of genotype data were missing and if the Hardy‐Weinberg equilibrium *P* < 10^−4^. To investigate population structure, principal components analysis was conducted^21^; the top‐10 principal components were retained to account for any ancestry differences in genetic structures that could bias results.^21^

#### *Polygenic Score*

To calculate PGS for Alzheimerʼs disease (AD‐PGS), we used summary statistics reported by the International Genomics of Alzheimerʼs Project (IGAP) (Supplementary Tables). AD‐PGS was calculated using methods previously described in the Health and Retirement Study.^23^ AD‐associated SNPs, weighted by their effect size derived from the IGAP,^24^ were summed in a continuous score using PRSice.^25^ Because previous research highlighted that PGSs built from directly genotyped data either had more predictive power^26^ or did not differ significantly from PGSs calculated using imputed data,^23^ we calculated PGSs based on genotyped data at different *P‐*value cutoffs. Because PGSs including all available SNPs either explain the most amount of variation in a trait or are not significantly different than PGSs based on other *P‐*value threshold^23^; we used PGS based on a *P*‐value threshold of 1. A total of 1,191,420 SNPs were included in AD‐PGS.

We also report results from a high‐resolution polygenic scoring approach implemented in PRSice.^25^ For this set of analyses, quality‐controlled SNPs were pruned using clumping to obtain SNPs in linkage equilibrium with an R^2^ = .1 within a 200‐bp window. PGSs were calculated at *P*‐value thresholds ranging from .001 to 1 (increments of .001); the best *P*‐value threshold was identified as the one that gave the smallest *P* value for association with clinical AD dementia.^25^ In the present study, the “best fit” *P*‐value threshold was .001 that encompassed 1,004 SNPs; we refer to this PGS as AD‐PGS_best‐fit_. To aid interpretability of the results, both AD‐PGS and AD‐PGS_best‐fit_ were standardized to a mean of 0 (standard deviation [SD] = 1).

### Statistical Analysis

#### *Imputation of Missing Values*

In the present study, some of the variables had up to 8% missing values (Supplementary Table S1). Given that complete case analysis can lead to bias,^27^ we imputed the missing values assuming missingness did not depend on unobserved values.^28^ For the imputation, we used missForest^29^ in RStudio v.3.6.0; this is an iterative imputation method based on Random Forests. The missForest was shown to outperform the well‐known imputation methods such as *k*‐nearest neighbors and parametric multivariate imputation by chained equations in the presence of nonlinearity and interactions.^29^ In the present study, the imputed values were closely aligned with the observed values for both continuous (normalized root mean squared error = .12%) and categories variables (proportion of falsely classified = .13%).^29^, ^30^

#### *Association Analyses and Interactions*

To investigate influences of PGSs on educational attainment and wealth on the time of dementia diagnosis, we used the accelerated failure time (AFT) survival model for right‐censored data. In contrast to Cox proportional hazards regression, the AFT does not assume that effects of the predictors on long‐term outcomes are constant over time, an assumption that may lead to biased parameter estimates.^31^ To identify the best fitting parametric model (ie, exponential, Weibull, lognormal, and gamma), we used the Akaike information criterion that showed the gamma model was the most appropriate for our analyses. The parameter coefficients from the AFT model were converted into mean difference in the time to dementia diagnosis through the equation ((e^β^ − 1) × mean time to diagnosis).^32^, ^33^ Positive values imply a longer time to conversion, and negative values imply a shorter time.

Interactions between PGSs and socioeconomic factors were investigated using multiplicative and additive models. The multiplicative model tests interaction as the departure from multiplicativity according to which the combined effect of risk factors differs from the product of their individual effects, whereas the additive interaction tests whether the combined effect of risk factors differs from the sum of their individual effects.^34^ The results from the additive interactions were derived using the relative excess risk due to interaction (RERI) and attributable proportion due to interaction (AP).^34^

#### *Sensitivity Analyses*

To examine whether the findings were applicable to all dementia or were specific to clinical AD dementia cases, we repeated the analyses, limiting them to either clinical AD dementia cases only or by removing individuals with a diagnosis of clinical AD dementia from the sample (non‐AD cases). Further, because PGS may be related to other brain‐related illnesses, we checked whether some participants in our sample had brain‐degenerative conditions such as Huntingtonʼs and Parkinsonʼs disease. Although none of our clinical AD dementia cases had these conditions, 60 (.89%) of the nondementia control group appeared to have them. Consequently, we re‐ran the analyses, additionally adjusting them for the presence of these conditions. Because beta estimates with corresponding confidence intervals (CIs) and *P* values did not change, we reported the original results. All association analyses were conducted in Stata v.16 (StataCorp LP, College Station, TX, USA).

## RESULTS

### Sample Characteristics

The sample comprised 7,039 individuals for whom the quality‐controlled genome‐wide genotyping and dementia status during follow‐up were available. Of these, 320 (4.6%) were classified as having dementia (ie, cases) over the 10‐year follow‐up, and 6,719 (95.4%) remained dementia free (ie, controls). Of all dementia cases, 76 (23.8%) had the diagnosis of clinical AD dementia, and 130 (40.6%) had *APOE*‐ε4. The baseline mean age for the entire sample was 64.8 years (SD = 9.4; median = 63; range = 50‐101). Among those diagnosed with dementia during follow‐up, the avenge time to dementia diagnosis was 8.2 years (SD = 3.3) (Table 1).

**Table 1:** Baseline Sample Characteristics of ELSA Participants

Baseline sample characteristics	Total sample N = 7,039	No‐dementia controls N = 6,719 (95.4%)	Dementia cases N = 320 (4.6%)	Test statistics		
	N (%)/Mean (SD)	N (%)/Mean (SD)	N (%)/Mean (SD)	*t*/*x* ^2^	*df*	*P* value
Age at baseline, y	64.8 (9.4)	64.3 (9.2)	73.8 (8.6)	−17.84	7,037	<.001
Male sex	3,254 (46.2)	3,120 (46.4)	134 (41.9)	2.56	1	.11
Age of dementia diagnosis (years)	‐	80.3 (8.5)	‐	‐	‐	‐
*APOE‐ε4* present	1,773 (25.2)	1,643 (24.4)	130 (40.6)	42.39	1	<.001
Currently smoker	1,023 (15.4)	981 (15.5)	42 (13.6)	.85	1	.35
Not married	4,832 (68.6)	4,642 (69.1)	190 (59.4)	13.39	1	<.001
Educational attainment	13.7 (3.8)	13.7 (3.8)	12.6 (3.5)	5.18	6,455	<.001
Wealth				22.75	2	<.001
Low	2,280 (33.3)	2,143 (32.8)	137 (43.4)			
Intermediate	2,285 (33.3)	2,177 (33.3)	108 (34.3)			
High	2,287 (33.4)	2,217 (33.9)	70 (22.2)			

Abbreviations: *APOE*‐ε4, two ε4 alleles of the apolipoprotein E gene; ELSA, English Longitudinal Study of Aging; SD, standard deviation.

**Figure 1:**
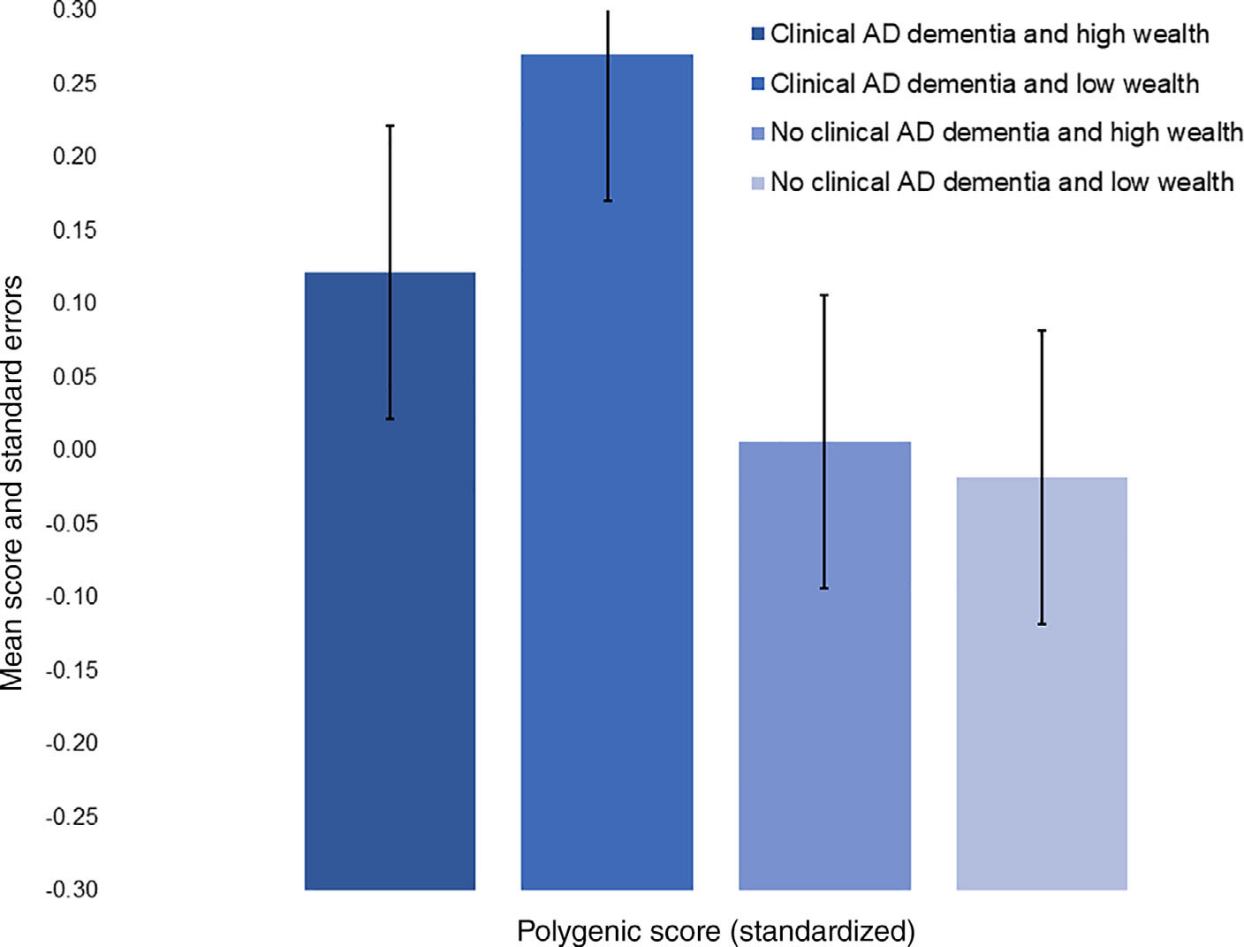
Mean polygenic score for Alzheimerʼs disease (AD) for different levels of wealth and presence of diagnosis of clinical AD dementia.

### PGS, Educational Attainment, and Wealth in Relation to Time of Dementia Diagnosis

A 1‐SD increase in AD‐PGS was associated with an average decrease in the time to dementia of 4.8 months (β = −.05; 95% CI = −.10 to −.001; *P* = .01) (Table 2). The presence of *APOE*‐ε4 was associated with an earlier dementia diagnosis by approximately 24.9 months (β = −.29; 95% CI = −.38 to −.20; *P* < .001). Independently from AD‐PGS, intermediate and low levels of wealth were shown to associate with an accelerated time to dementia diagnosis of 12.0 months and 18.7 months, respectively (Table 2). When using AD‐PGS_best‐fit_ (Supplementary Table S2), 1‐year increase in completed schooling was associated with the decelerated time to dementia diagnosis of 2.0 months (β = .02; 95% CI = .002‐.03; *P* = .04) but not in a model with AD‐PGS. Neither multiplicative nor additive interaction models yielded significant interactions between AD‐PGS, and AD‐PGS_best‐fit_, and socioeconomic factors in association with the time of dementia diagnosis (Supplementary Tables S2 and S3). When individuals with clinical AD dementia were removed from the analyses (Supplementary Table S4), a 1‐SD increase in AD‐PGS was associated with a mean decrease in the time to non‐AD diagnosis of 5.8 months (β = −.06; 95% CI = −.11 to −.01; *P* = .02). Independently from AD‐PGS, a low level of wealth was associated with an accelerated time to non‐AD diagnosis by an average of 24.1 months. No significant interaction effects were found between AD‐PGS and AD‐PGS_best‐fit_ (Supplementary Table S5) and socioeconomic factors in association with the time of non‐AD diagnosis.

**Table 2:** Multivariate Accelerated Failure Time Model Estimating Difference in Time to Diagnosis of Dementia in Older Adults during the 10‐Year Follow‐Up

Dementia		Variables	Multiplicative interaction effect			
			β	95% CI	*P* value	β converted to time to diagnosis
PGS score only		PGS	−.05	−.10 to −.01	.01	−4.82
		*APOE*‐ε4	−.29	−.38 to −.20	<.001	−24.86
Educational attainment	Main effect	PGS	−.12	−.28 to .04	.15	−11.17
		*APOE*‐ε4	−.29	−.38 to −.20	<.001	−24.86
		Educational attainment	.01	.001 to .02	.08	.99
	Interaction	PGS * Educational attainment	.01	−.01 to .02	.42	.99
Wealth	Main effect	PGS	−.001	−.09 to .09	.98	−.10
		*APOE*‐ε4	−.29	−.38 to −.20	<.001	−24.86
		High	‐	‐	‐	‐
		Intermediate	−.13	−.24 to −.01	.03	−12.04
		Low	−.21	−.30 to −.07	<.001	−18.71
	Interaction	PGS * High	‐	‐	‐	‐
		PGS * Intermediate	−.04	−.15 to .03	.49	−3.87
		PGS * Low	−.09	−.20 to .02	.13	−8.50

Note: The *β* coefficients in the AFT model were converted into time to first dementia diagnosis through the equation: ((e^β^ − 1) × mean time to dementia diagnosis). All analyses are adjusted for age, sex, marital status, current smoking status, *APOE*‐ε4, and four genetic principal components. The asterisk indicates an interaction between two variables.

Abbreviations: *APOE*‐ε4, two ε4 alleles of the apolipoprotein E gene; CI, confidence interval; PGS, polygenic score.

### PGS, Educational Attainment, and Wealth in Relation to Timing of Clinical AD Dementia Diagnosis

Although AD‐PGS was not significantly associated with clinical AD dementia, the presence of *APOE*‐ε4 in participants diagnosed with clinical AD dementia was associated with an accelerated timing of clinical AD dementia diagnosis by approximately 33.9 months. A 1‐year increase in schooling was associated with decelerated time to clinical AD dementia diagnosis (β = .03; 95% CI = .01‐.06; *P* = .01) entailing a mean increase in the time to development of clinical AD dementia diagnosis of 3.0 months (Table 3). The interaction as the departure from multiplicativity highlighted a significant interaction effect between AD‐PGS and educational attainment (β = .03; 95% CI = .01‐.06; *P* = .01) corresponding to a decelerated time to clinical AD dementia diagnosis by 3.0 months. A significant multiplicative interaction effect between AD‐PGS and intermediate (β = −.28; 95% CI = −.49 to −.06; *P* = .01) and low (β = −.24; 95% CI = −.45 to −.02; *P* = .03) levels of wealth in association with the time to clinical AD dementia diagnosis highlighted that a 1‐SD increase in AD‐PGS was associated with an accelerated timing of clinical AD dementia diagnosis by 24.1 months in participants with an intermediate level of wealth and 21.1 months in participants with low wealth (Figure 1). There also was a significant additive interaction between AD‐PGS and an intermediate level of wealth (RERI = −.26; 95% CI = −.27 to −.06; AP = −.35; 95% CI = −.62 to −.04) (Supplementary Table S6). Neither multiplicative nor additive interaction models yielded significant interactions between AD‐PGS_best‐fit_ and socioeconomic factors in association with the time of clinical AD dementia diagnosis (Supplementary Table S7).

**Table 3:** Multivariate Accelerated Failure Time Model Estimating difference in Time to the Diagnosis of Clinical AD Dementia during the 10‐Year Follow‐Up

Dementia		Variables	Multiplicative interaction effect			β converted to time to diagnosis
				95% CI	*P* value		
PGS score only		PGS	−.06	−.14 to .02	.12	−5.75
		*APOE*‐ε4	−.42	−.60 to −.23	<.001	−33.87
Educational attainment	Main effect	PGS	−.47	−.78 to −.17	.01	−59.25
		*APOE*‐ε4	−.41	−.58 to −.23	<.001	−33.22
		Educational attainment	.03	.01 to .06	.01	3.01
	Interaction	PGS * Educational attainment	.03	.01 to .06	.01	3.01
Wealth	Main effect	PGS	.13	−.02 to .29	.10	13.71
		*APOE*‐ε4	−.41	−.60 to −.23	<.001	−33.22
		High	‐	‐	‐	‐
		Intermediate	−.13	−.33 to .08	.22	13.71
		Low	−.10	−.31 to .12	.35	−9.40
	Interaction	PGS * High	‐	‐	‐	
		PGS * Intermediate	−.28	−.49 to −.06	.01	−24.12
		PGS * Low	−.24	−.45 to −.02	.03	−21.07

Note: The *β* coefficients in the accelerated failure time model were converted into time to first dementia diagnosis through the equation ((e^β^ − 1) × mean time to dementia diagnosis). All analyses are adjusted for age, sex, marital status, current smoking status, *APOE*‐ε4, and four genetic principal components. The asterisk indicates an interaction between the two variables.

Abbreviations: AD, Alzheimerʼs disease; CI, confidence interval; PGS, polygenic score.

## DISCUSSION

To our knowledge, this is the first study to have investigated interactions between AD‐PGS and socioeconomic factors, such as educational attainment and accumulated financial wealth, in predicting and quantifying the time to dementia diagnosis and dementia subgroups, independently from effects of the *APOE*‐ε4 status in a large population‐representative sample of older adults.

Our results indicate that a higher aggregate of loci for AD may exert its effect by accelerating the clinical presentation of dementia, with illness diagnosis approximately 4.8 months earlier in older adults with high polygenic risk than for those with lower PGS. When AD cases were removed from the analyses, higher PGS was associated with an earlier non‐AD diagnosis by an average of 5.8 months. These findings confirm that dementia has a strong polygenic architecture.^12^, ^24^ However, using PGS calculated from a subset of genetic markers after pruning out SNPs in linkage disequilibrium and applying a *P*‐value threshold did not yield significant associations between higher genetic load of multiple alleles and the time to dementia diagnosis. The differences in the number of genetic variants included in each PGS depending on computational methods may explain these findings. PGS calculated from hundreds to thousands of common variants arguably captures the causative variants^35^; thus in the present study, having included 1.2 M common loci associated with AD, AD‐PGS may represent the true genetic risk. However, such a genetic score may accumulate noise and thus may lead to false associations.^36^ In contrast, creating polygenic profiling based on pruning and *P*‐value threshold was criticized for discarding potentially important information and limiting prediction accuracy,^37^, ^38^ potentially leading to negative findings.

Although a significant association between PGS and the age of AD diagnosis was highlighted,^39^, ^40^ we did not find a significant relationship between AD‐PGS and the time to clinical AD dementia diagnosis in our sample. Whereas in previous studies PGS was based on either 10 AD risk variants^39^ or only those variants that reached a genome‐wide association study (GWAS) significance level,^40^ PGS used in the present study was not restricted to any loci specific to AD nor to GWAS‐significant variants. Therefore, a potentially significant association between AD‐PGS and the time to clinical AD dementia diagnosis might have been masked by the inclusion of an excessive number of genetic variants. Nonetheless, the nonsignificant association remained when PGS was calculated based on a much smaller number of SNPs selected through high‐resolution scoring adjusting for linkage disequilibrium. It is also feasible that this negative finding may reflect the relatively small sample of clinical AD dementia cases available in the ELSA cohort. Moreover, *APOE*‐ε4 was associated with an accelerated time to dementia diagnosis by approximately 24.9 months and 33.9 months for clinical AD dementia diagnosis. This finding supports the notion that the predictive validity for PGSs is not yet on a par with *APOE*‐ε4^41^ in estimating the time to dementia and clinical AD dementia diagnosis.

A higher education attainment is thought to protect against dementia risk.^7^, ^8^ Our results estimated that each year of completed schooling was associated with a delayed diagnosis of clinical AD dementia of approximately 3.0 months, but this association was nonsignificant for dementia diagnosis. Further, 1 year of completed schooling was associated with a delayed diagnosis of clinical AD dementia by approximately 3.0 months among individuals at higher genetic risk. This suggests that educational attainment may serve as a protective mechanism against the illness among older people with higher polygenic risk. In support of the social determinants hypothesis,^42^ according to which educational attainment attenuates the role played by genetic risk factors, our findings show that the effects of education on the timing of the diagnosis of the illness are not consistent across dementia subtypes, benefiting more those with a diagnosis of clinical AD dementia and greater polygenic risk.

Lower wealth, which may reflect limited socioeconomic resources, low digital literacy, and limited access to participation in cultural activities or reduced social networks, is an important factor in dementia diagnosis.^6^ We showed that one of the pathways through which low wealth exerts its effect may be by accelerating the clinical presentation of dementia leading to an earlier illness diagnosis by approximately 12.0 to 18.9 months independently from AD‐PGS and *APOE*‐ε4. In relation to clinical AD dementia diagnosis, lower levels of wealth appear to interact with AD‐PGS by accelerating the time to clinical AD dementia diagnosis by approximately 21.1 to 24.1 months compared with older people with a low genetic loading and a high level of accumulated wealth. This is consistent with the genetic liability threshold model according to which the combined effect of many genetic risk variants with other factors causes an individual to cross the threshold leading to the development of the condition.^43^ These results hold promise for preventive strategies aiming to delay the first diagnosis of clinical AD dementia.

### Strengths and Limitations

We analyzed a large population‐based cohort who are nationally representative of older adults in England. Our study also included a relatively equal proportion of women and men from socioeconomically diverse backgrounds. We benefited from more detailed assessments of wealth than are available in most current studies because this measure was computed based on information on multiple individual components rather than on the broad categorization of assets. Nonetheless, using a doctorʼs diagnosis to identify most dementia and clinical AD dementia cases may mean that our study underestimated dementia cases and that the timing of diagnosis may not be entirely accurate. Because of the relatively small number of dementia cases, we could not explore the types of dementia in detail, stratify analyses by age, and add additional models investigating the competing risk of *APOE* and heart disease in relation to the time of dementia diagnosis, as this may increase likelihood of false results due to multiple testing.

Similarly, to avoid overfitting, we also were unable to adjust our models for interactions between the covariates as advised.^44^ Although PGSs have a potential to improve health outcomes through their eventual routine implementation as clinical biomarkers, the poor generalizability of genetic studies across populations is noteworthy.^45^ This is because the construction of PGSs depends largely on the availability of the summary statistics from GWAS. However, approximately 79% of all GWAS participants are of European descent despite making up only 16% of the global population.^45^ Given that genetic risk is different in European and non‐European individuals, further work is necessary to develop a PGS model in nonwhite populations.

In conclusion, PGS provides a strong tool for prediction of the length of time to dementia and clinical AD dementia diagnosis. Educational attainment and low wealth appear to be important factors influencing the time to the illness in older individuals, particularly those with higher AD‐PGS. Although these findings need to be replicated in an independent sample with larger numbers of dementia or clinical AD dementia cases, they demonstrate that public health strategies for dementia prevention should protect those who are socioeconomically disadvantaged and have a higher PGS.

## Supporting information


**Supplementary Appendix S1**: Supplementary Material.
**Supplementary Table S1**: Distribution of missing and observed variables at baseline and follow‐up in the English Longitudinal Study of Aging.
**Supplementary Table S2**: Multivariate Accelerated Failure Time model estimating difference in time to the first diagnosis of all‐cause dementia in older adults during the 10‐year follow‐up in association with AD‐PGSbest‐fit calculated using the software PRSice.
**Supplementary Table S3**: Multivariate Accelerated Failure Time model estimating difference in time to diagnosis of dementia in older adults during the 10‐year follow‐up.
**Supplementary Table S4**: Multivariate Accelerated Failure Time model estimating difference in time to diagnosis of dementia, excluding cases with clinical Alzheimerʼs disease, in older adults during the 10‐year follow‐up.
**Supplementary Table S5**: Multivariate Accelerated Failure Time model estimating difference in time to diagnosis of dementia excluding the cases with clinical Alzheimerʼs disease (AD) during the 10‐year follow‐up in association with AD‐PGSbest‐fit calculated using the software PRSice.
**Supplementary Table S6**: Multivariate Accelerated Failure Time model estimating difference in time to the diagnosis of clinical Alzheimerʼs dementia during the 10‐year follow‐up.
**Supplementary Table S7**: Multivariate Accelerated Failure Time model estimating difference in time to diagnosis of Alzheimerʼs disease (AD) during the 10‐year follow‐up in association with AD‐PGSbest‐fit calculated using the software PRSice.
