# Supplementary material for: Interplay between Socioeconomic Markers and Polygenic Predisposition on Timing of Dementia Diagnosis
Source: J Am Geriatr Soc. 2020 Mar 18;68(7):1529–36. doi: 10.1111/jgs.16406 (PMC7363562; doi:10.1111/jgs.16406)
Supplement: Supplementary file 1 — Supplementary Appendix S1: Supplementary Material. Supplementary Table S1: Distribution of missing and observed variables at baseline and follow‐up in the English Longitudinal Study of Aging. Supplementary Table S2: Multivariate Accelerated Failure Time model estimating difference in time to the first diagnosis of all‐cause dementia in older adults during the 10‐year follow‐up in association with AD‐PGSbest‐fit calculated using the software PRSice. Supplementary Table S3: Multivariate Accelerated Failure Time model estimating difference in time to diagnosis of dementia in older adults during the 10‐year follow‐up. Supplementary Table S4: Multivariate Accelerated Failure Time model estimating difference in time to diagnosis of dementia, excluding cases with clinical Alzheimerʼs disease, in older adults during the 10‐year follow‐up. Supplementary Table S5: Multivariate Accelerated Failure Time model estimating difference in time to diagnosis of dementia excluding the cases with clinical Alzheimerʼs disease (AD) during the 10‐year follow‐up in association with AD‐PGSbest‐fit calculated using the software PRSice. Supplementary Table S6: Multivariate Accelerated Failure Time model estimating difference in time to the diagnosis of clinical Alzheimerʼs dementia during the 10‐year follow‐up. Supplementary Table S7: Multivariate Accelerated Failure Time model estimating difference in time to diagnosis of Alzheimerʼs disease (AD) during the 10‐year follow‐up in association with AD‐PGSbest‐fit calculated using the software PRSice. [file JGS-68-1529-s001.pdf]

Supplementary Material

Olesya Ajnakina, PhD [1,2 *], Dorina Cadar, PhD [1], Andrew Steptoe, DSc [1]

[1] Department of Behavioural Science and Health, Institute of Epidemiology and Health Care, University College London, London, United Kingdom

[2] Department of Biostatistics & Health Informatics, Institute of Psychiatry, Psychology and Neuroscience, King's College London, London, United Kingdom

*Corresponding author: Olesya Ajnakina, Department of Behavioural Science and Health, Institute of Epidemiology and Health Care, University College London, 1-19 Torrington Place, London, WC1E 7HB, United Kingdom; Email: o.ajnakina@ucl.ac.uk

Supplementary Methods

Study participants

The English Longitudinal Study of Ageing (ELSA) is a large, multidisciplinary study of cohort of men and women living in England aged 50 year older and over and who are representative of the English population both in terms of socioeconomic profile and geographic region.[1] The study commenced in 2002 and the cohort was then followed-up every two years, with periodic refreshments to maintain the age profile. Since 2002 there have been 8 waves of data collection providing detailed information on health, well-being and socioeconomic circumstances. Further, the ELSA study has been modelled on the US Health and Retirement Study (HRS).[2] This was done to facilitate harmonisation with across the international family of HRS longitudinal studies, and thus to promote international comparisons in the age-related outcomes across the population-based cohorts.

Consent and Administration Procedures

The ELSA participants were eligible for blood data collection if they had successfully completed the nurse visit and gave consent for blood samples to be taken. The respondents were not eligible to have a blood sample taken if they: 1) had a clotting or bleeding disorder, 2) ever had a fit or convulsion, 3) were taking anticoagulant drugs (such as Warfarin, Protamine or Acenocoumarol), or 4) were pregnant. If the ELSA participants were eligible to have a blood sample, nurses then determined whether they were eligible to fast. Those respondents who were determined to be eligible to fast, were instructed not eat, smoke, drink alcohol or do any vigorous exercise 30 minutes before giving the blood sample. The responders were exempted from fasting if they: 1) were aged 80 or over, 2) were diabetic and on treatment, or 3) were malnourished or otherwise unfit to fast (as judged by the nurse). All respondents could still drink water and take their medication as normal.

Genotyping Process

The genome-wide genotyping was performed at University College London (UCL) Genomics in 2013-2014 with the funding the Economic and Social Research Council (ESRC). This involved genotyping of 7,597 ELSA participants of European ancestry using the llumina HumanOmni2.5 BeadChips (HumanOmni2.5-4v1, HumanOmni2.5-8v1.3), which measures ~2.5 million markers that capture the genomic variation down to 2.5% minor allele frequency (MAF). Genotyping was performed in two batches. Allele frequencies were compared between the batches after filtering for 5% of missingness. The correlation was calculated between the batches for a number of chromosomes and exceeded 99%. The two batches were merged as one data set. After quality reassurance, which entailed excluding ethnic outliers (self-reported) and duplicates, the genome-wide data were available for total 7412 ELSA participants of European ancestry and 2230767 SNPs.

GWAS Quality Control

Before the GWAS data was utilised for creating PGSs, a thorough quality control (QC)[3] at both individuals and single-nucleotide polymorphism (SNPs) levels was carried out using Quality control was performed using PLINK 1.9,[4] R,[5] and VCFtools.[6]

QC based on individual level. The samples for whom the recorded sex phenotype was inconsistent with genetic sex were removed. Duplicated samples and cryptic relatedness between each pair of participants was evaluated using pairwise genome-wide estimates of three coefficients corresponding to the probabilities of sharing 0, 1 or 2 alleles between two individuals that are identical by descent.[7] There are two methods for estimating the identical-by-descent (IBD) probabilities - method of moments and method of maximum likelihood. Both methods have been shown to give very similar results;[8] thus we report results from method of moments implemented in PLINK 1.9.[4] IBD were estimated using autosomal SNPs where IBD=1 highlights presence of duplicates or monozygotic twins, IBD=0.5 shows that first-degree relatives are present in the sample, IBD=0.25 and IBD=0.125 highlights presence of

second-degree and third-degree relatives, respectively.[9] Owing to genotyping error, linkage disequilibrium (LD) and population structure, it is expected to observe some variations around these theoretical values. Therefore, it is normal to remove one individual from each pair with an IBD value of >0.2, which is halfway between the expected IBD for third- and second-degree relatives.[3] We identified individuals with an IBD value of >0.2 and excluded one of each pair at random.

QC based on SNP level. Heterozygosity refers to carrying of two different alleles of a specific SNP. Excessive heterozygosity may imply a sample contamination, while less heterozygosity than expected may imply inbreeding.[3] In the ELSA study checks for heterozygosity were performed on a set of SNPs which were non-(highly) correlated. To generate a list of non-(highly) correlated SNPs, we excluded regions that are known to contain clusters of highly correlated SNPs. These were the Lactase Gene (LCT) (chromosome 6, 12578740-135837195 bp), human leukocyte antigen (HLA) (chromosome 2, 2550000-3350000 bp) and two inversion regions located on 8p23.1 (chromosome 8, 81305000-1200000 bp) and 17q21.31 (chromosome 17, 40900000-45000000 bp),[10] major histocompatibility complex (MHC) (chromosome 6: 26000000-3000,000 bp), and *APOE* (chromosome 19: 45116911-46318605 bp).[11] We then pruned the SNPs using the '10 5 0.1' parameters. These pruning parameters use a sliding window method that considers blocks of 10 SNPs and removes SNPs with r^2 >0.10 afterward shifting the window by 5 SNPs. Those individuals with extremely low or high heterozygosity score (>3 standard deviations from the mean) were removed. Further, the genotyped data with a call rate of <98% was removed. SNPs in sex chromosomes and SNPs with a minor allele frequency (MAF) of <0.01 were excluded. SNPs whose genotype distributions deviated significantly from the Hardy-Weinberg equilibrium (HWE) ($p<10^{-4}$) and with missingness <0.02 were also removed.

Population structure. To investigate population structure, we use principal components analysis (PCA)[12] implemented in PLINK 1.9. We used PCA approach with two aims; first, to identify those individuals who deviate from the ethnic population they self-reported to be (i.e., ethnic outliers), and second, to provide sample eigenvectors which will then be used for adjusting for possible population stratification in the association analyses.[12,13] It has been shown that in PCA, the usefulness of certain principal components

(PCs) may be limited by clusters of highly correlated SNPs at specific locations, such as the LCT, HLA, 8p23.1 and 17q21.31, MHC, and APOE[8,10] in whole-genome arrays.[11] To address this pitfall, the SNPs that were used in PCA were selected by LD pruning from an initial pool consisting of all autosomal SNPs with a missing call rate <5% and MAF >5%. In addition, the 2q21 (LCT), HLA, 8p23, and 17q21.31, MHC, and APOE regions were excluded from this initial pool. The LD pruning process, using all unrelated ELSA participants selected 154471 autosomal SNPs with all pairs having r^2<0.1 in a sliding 10 Mb window. PCs were obtained using PLINK1.9;[4] we retained the top 10 PCs to account for any ancestry differences in genetic structures that could bias results.[12] Initially, we performed PCA on all study subjects; however, the visual inspection of the PCs distribution highlighted the present of ancestral admixture in the 65 individuals. We removed these outliers and re-calculated PCs using the updated samples.

Polygenic scores (PGS)

Polygenic scores (PGS) can be defined as a single value estimate of an individual's propensity to a phenotype, calculated as a sum of their genome-wide genotypes weighted by corresponding genotype effect sizes from GWAS summary statistics.[14,15] For PGS used in the present study, we used the summary statistics from stage 1 genome-wide association studies (GWAS) on individuals of European ancestry reported by the International Genomics of Alzheimer's Project (IGAP). [16] In stage 1, IGAP used genotyped and imputed data on 7055881 single nucleotide polymorphisms (SNPs) to meta-analyse four previously-published GWAS datasets consisting of 17008 Alzheimer's disease cases and 37154 controls (The European Alzheimer's disease Initiative - EADI the Alzheimer Disease Genetics Consortium - ADGC The Cohorts for Heart and Aging Research in Genomic Epidemiology consortium - CHARGE The Genetic and Environmental Risk in AD consortium - GERAD).

PGS was then calculated for each individual in the target sample following the formula outlined below:

$$\mathrm{PGS}^{i} = \sum_{j=1}^{j} W^{j} G^{ij}$$

where *i* is individual *i* (i=1 to *N*), *j* is SNP *j* (j=1 to *J*), *W* is the meta-analysis effect size for SNP *j* and *G* is the genotype, or the number of reference alleles (0, 1, or 2), for individual *i* at SNP *j*. The profile score is then evaluated through regression of the target sample phenotype on the PGS after accounting for other known covariates.

Because SNP effects are estimated with some uncertainty and not all SNPs influence the trait under study, PGS are calculated at different pre-specified significance threshold of quality controlled and autosomal SNPs.[14] This in turn allows testing associations with the target trait for each threshold and thus optimising the prediction. Accordingly, we performed PGSs based on threshold of *p*-values of 0.001, 0.01, 0.05, 0.1, 0.3, and 1 employing methodology as originally described by Health and Retirement Study (HRS).[14,17] Nonetheless, the HRS team examined four traits with large published and replicated GWASs on four phenotypes (i.e., height, body mass index, educational attainment, and depression) demonstrating that PGSs that included all available SNPs either explained the most amount of variation in an outcome or were not significantly different than the PGSs that did. Therefore, in the present study we utilised the PGS that was based on threshold of *p*-value of 1 employing methodology as originally described.[14,17] Furthermore, we built PGSs based on the directly genotyped data rather than imputed data. This decision was based on the previous research findings which highlighted that the PGSs built from the directly genotyped data had more predictive power[18] or did not differ significantly from the PGSs that were based on imputed data.[17] These analyses were performed using PRSice[19], and PLINK 1.9.[4]

REFERENCES:

1. Steptoe A, Breeze E, Banks J, Nazroo J. Cohort profile): the English longitudinal study of ageing. Int J Epidemiol 2013; 42: 1640-8.

2. Sonnega A, Faul JD, Ofstedal MB, Langa KM, Phillips JW, Weir DR. Cohort Profile: the Health and Retirement Study (HRS). Int J Epidemiol 2014; 43: 576-85.

3. Marees AT, de Kluiver H, Stringer S, Vorspan F, Curis E, Marie-Claire C, Derks EM. A tutorial on conducting genome-wide association studies: Quality control and statistical analysis. Int J Methods Psychiatr Res 2018; 27: 1-10.

4. Chang CC, Chow CC, Tellier LC, Vattikuti S, Purcell SM, Lee JJ. Second-generation PLINK: rising to the challenge of larger and richer datasets. Gigascience 2015; 4: 7.

5. RStudioTeam. RStudio: Integrated Development for R. Inc, Boston, MA 2016; URL http://www.rstudio.com/.

6. Danecek P, Auton A, Abecasis G, et al. The variant call format and VCFtools. Bioinformatics 2011; 27: 2156-8.

7. Huff CD, Witherspoon DJ, Simonson TS, Xing J, Watkins WS, Zhang Y, et al. Maximum-likelihood estimation of recent shared ancestry (ERSA). Genome Res 2011; 21: 768-74.

8. Laurie CC, Doheny KF, Mirel DB, Pugh EW, Bierut LJ, Bhangale T, et al. Quality control and quality assurance in genotypic data for genome-wide association studies. Genet Epidemiol 2010; 34: 591-602.

9. Anderson CA, Pettersson FH, Clarke GM, Cardon LR, Morris AP, Zondervan KT. Data quality control in genetic case-control association studies. Nat Protoc 2010; 5: 1564-73.

10. Novembre J, Johnson T, Bryc K, Kutalik Z, Boyko AR, Auton A, et al. Genes mirror geography within Europe. Nature 2008; 456: 98-101.

11. Kunkle BW, Grenier-Boley B, Sims R, et al. Genetic meta-analysis of diagnosed Alzheimer's disease identifies new risk loci and implicates Abeta, tau, immunity and lipid processing. Nat Genet 2019; 51: 414-30.

12. Price AL, Patterson NJ, Plenge RM, Weinblatt ME, Shadick NA, Reich D. Principal components analysis corrects for stratification in genome-wide association studies. Nat Genet 2006; 38: 904-9.

13. Wang D, Sun Y, Stang P, Berlin JA, Wilcox MA, Li Q. Comparison of methods for correcting population stratification in a genome-wide association study of rheumatoid arthritis: principal-component analysis versus multidimensional scaling. BMC Proc 2009; 3: S109.

14. International Schizophrenia Consortium, Purcell SM, Wray NR, Stone JL, Visscher PM, O'Donovan MC, et al. Common polygenic variation contributes to risk of schizophrenia and bipolar disorder. Nature 2009; 460: 748-52.

15. Choi SW, Mak, TSH, O'Reilly P. A guide to performing Polygenic Risk Score analyses. BioRxiv 2018; 1-22.

16. Lambert JC, Ibrahim-Verbaas CA, Harold D, et al. Meta-analysis of 74,046 individuals identifies 11 new susceptibility loci for Alzheimer's disease. Nat Genet 2013; 45: 1452-8.

17. Ware EB, et al. Method of Construction Affects Polygenic Score Prediction of Common Human Trait. BiorXiv 2017; 1-13.

18. Okbay A, Beauchamp JP, Fontana MA, Lee JJ, Pers TH7, Rietveld CA, et al. Genome-wide association study identifies 74 loci associated with educational attainment. Nature 2016; 533: 539-42.

19. Euesden J, Lewis CM, O'Reilly PF. PRSice: Polygenic Risk Score software. Bioinformatics 2015; 31: 1466-8.

Supplementary Results

Supplementary Table S1. Distribution of missing and observed variables at baseline and follow-up in ELSA

Variables at baseline	N observed	% observed	N missing	% missing
Age	7039	100	0	0.00
Clinical AD dementia	6795	96.53	244	3.47
APOE-ε4	7039	100	0	0.00
Current smoking status	6643	94.37	396	5.63
Educational attainment	6457	91.73	582	8.27
Sex	7039	100	0	0.00
Levels of wealth	6852	97.34	187	2.66
Time to the illness diagnosis	7039	100	0	0.00
Married	7039	100	0	0.00
Non-AD dementia	6963	98.92	76	10.8

AD, Alzheimer's disease; *APOE-ε4,* Apolipoprotein E gene with the two ε4 alleles

Supplementary Table S2. Multivariate Accelerated Failure Time model estimating difference in time to the first diagnosis of all-cause dementia in older adults during the 10-year follow-up in association with AD-PGS$_{best\text{-}fit}$ calculated using the software PRSice

			Additive interaction effect		Multiplicative interaction effect			
Dementia		Variables	RERI (95% CI)	AP (95% CI)	β (SE)	95% CI	p-value	β converted to time to dementia diagnosis
PGS score only		PGS	-	-	-0.01 (0.03)	-0.06-0.04	.78	-0.99
		APOE	-	-	-0.29 (0.06)	-0.41- -0.18	<.001	-24.86
Educational attainment	Main effect	PGS	-	-	0.05 (0.08)	-0.10-0.21	.51	4.82
		APOE	-	-	-0.28 (0.06)	-0.40- -0.17	<.001	-24.12
		Educational attainment	-	-	0.02 (0.01)	0.002-0.03	.04	1.96
	Interaction	PGS * Educational attainment	-0.01 (-0.02-0.20)	-0.01 (-0.02-0.11)	-0.01 (0.01)	-0.02-0.07	.42	-0.99
Wealth	Main effect	PGS	-		-0.03 (0.05)	-0.12-0.06	.56	-3.01
		APOE	-	-	-0.29 (0.06)	-0.40- -0.17	<.001	-24.86
		High	-	-	-	-	-	-
		Intermediate	-	-	-0.14 (0.06)	-0.26- -0.01	.03	-12.90
		Low	-	-	-0.21 (0.06)	-0.33- -0.09	.00	-18.71
	Interaction	PGS * High	-	-	-	-	-	-
		PGS * Intermediate	0.01 (-0.05-0.12)	0.01 (-0.07-0.10)	0.002 (0.06)	-0.11-0.11	.97	0.20
		PGS * Low	0.04 (-0.01-0.15)	0.05 (-0.01-0.13)	0.04 (0.05)	-0.06-0.15	.42	3.87

Effect size is indicated by *β* coefficient and standard error (SE) from the accelerated failure time survival model. CI, confidence interval; AD, Alzheimer's disease; PGS, polygenic score.

All analyses are adjusted for age, gender, marital status, current smoking status, *APOE*-ε4 and 4 genetic principal components.

PGS, polygenic score; RERI, relative excess risk due to interaction; AP, attributable proportion; RERI=0; AP=0: no interaction or exactly equal to additivity of the individual effects of the two risk factors; RERI>0; AP>0: positive interaction or more than additivity of the individual effects of the two risk factors; RERI<0; AP<0: negative interaction or less than additivity of the individual effects of the two risk factors.

β coefficients in the AFT model were converted into time to first dementia diagnosis through the equation: $((e^{\beta}-1) \times$ mean time to dementia diagnosis).

Supplementary Table S3. Multivariate Accelerated Failure Time model estimating difference in time to diagnosis of dementia in older adults during the 10-year follow-up

			Additive interaction effect	
Dementia		Variables	RERI (95%CI)	AP (95%CI)
PGS score only		PGS	-	-
		APOE-ε4	-	-
Educational attainment	Main effect	PGS	-	-
		APOE-ε4	-	-
		Educational attainment	-	-
	Interaction	PGS * Educational attainment	0·01 (-0·01-0·01)	0·01 (-0·01-0·01)
Wealth	Main effect	PGS	-	-
		APOE-ε4	-	-
		High	-	-
		Intermediate	-	-
		Low	-	-
	Interaction	PGS * High	-	-
		PGS * Intermediate	-0·03 (-0·08-0·03)	-0·04 (-0·13-0·03)
		PGS * Low	-0·07 (-0·10-0·01)	-0·09 (-0·18-0·01)

Effect size is indicated by β coefficient and standard error (SE) from the accelerated failure time survival model; CI, confidence interval.

All analyses are adjusted for age, sex, marital status, current smoking status, *APOE*-ε4 and 4 genetic principal components.

PGS, polygenic score; RERI, relative excess risk due to interaction; AP, attributable proportion; RERI=0; AP=0: no interaction or exactly equal to additivity of the individual effects of the two risk factors; RERI>0; AP>0: positive interaction or more than additivity of the individual effects of the two risk factors; RERI<0; AP<0: negative interaction or less than additivity of the individual effects of the two risk factors.

β coefficients in the AFT model were converted into time to first dementia diagnosis through the equation: $((e^{\beta}-1)\times$ mean time to dementia diagnosis)

Supplementary Table S4. Multivariate Accelerated Failure Time model estimating difference in time to diagnosis of dementia, excluding cases with clinical AD dementia, in older adults during the 10-year follow-up

			Additive interaction effect		Multiplicative interaction effect			
Dementia		Variables	RERI (95% CI)	AP (95% CI)	β (SE)	95% CI	p-value	β converted to time to dementia diagnosis
PGS score only		PGS	-	-	-0.06 (0.03)	-0.11- -0.01	.02	-5.75
		APOE	-	-	-0.25 (0.05)	-0.36- -0.15	<.001	-21.85
Educational attainment	Main effect	PGS	-	-	-0.01 (0.10)	-0.20-0.17	.87	-0.99
		Educational attainment	-	-	0.01 (0.01)	-0.01-0.02	.22	0.99
		APOE	-	-	-0.25 (0.05)	-0.36- -0.15	<.001	-21.85
	Interaction	PGS * Educational attainment	0.00 (-0.01-0.02)	-0.001 (-0.02-0.01)	-0.004 (0.01)	-0.02-0.01	.64	-0.39
Wealth	Main effect	PGS	-	-	-0.07 (0.06)	-0.18-0.05	.26	-6.68
		APOE	-	-	-0.26 (0.05)	-0.36- -0.15	<.001	-22.61
		High	-	-	-	-	-	-
		Intermediate	-	-	-0.14 (0.07)	-0.29-0.01	.06	-12.90
		Low	-	-	-0.28 (0.07)	-0.42- -0.13	<.001	-24.12
	Interaction	PGS * High	-	-	-	-	-	-
		PGS * Intermediate	0.07 (-0.01-0.25)	0.08 (-0.01-0.19)	0.07 (0.07)	-0.08-0.21	.37	6.68
		PGS * Low	0.00 (-0.18-0.10)	-0.01 (-0.27-0.10)	-0.03 (0.07)	-0.16-0.11	.68	-2.92

Effect size is indicated by β coefficient and standard error (SE) from the accelerated failure time survival model; CI, confidence interval; AD, Alzheimer's disease; PGS, polygenic score.

All analyses are adjusted for age, gender, marital status, current smoking status, *APOE*-ε4 and 4 genetic principal components.

PGS, polygenic score; RERI, relative excess risk due to interaction; AP, attributable proportion; RERI=0; AP=0: no interaction or exactly equal to additivity of the individual effects of the two risk factors; RERI>0; AP>0: positive interaction or more than additivity of the individual effects of the two risk factors; RERI<0; AP<0: negative interaction or less than additivity of the individual effects of the two risk factors.

β coefficients in the AFT model were converted into time to first dementia diagnosis through the equation: $((e^{\beta}-1) \times$ mean time to dementia diagnosis$)$

Supplementary Table S5. Multivariate Accelerated Failure Time model estimating difference in time to diagnosis of dementia excluding the cases with clinical AD dementia during the 10-year follow-up in association with AD-$PGS_{best\text{-}fit}$ calculated using the software PRSice

			Additive interaction effect		Multiplicative interaction effect			
Dementia		Variables	RERI (95% CI)	AP (95% CI)	β (SE)	95% CI	p-value	β converted to time to dementia diagnosis excluding cases with clinical AD dementia
PGS score only		PGS	-	-	-0.004 (0.03)	-0.06-0.06	.91	
		APOE	-	-	-0.26 (0.07)	-0.40- -0.13	<.001	-22.61
Educational attainment	Main effect	PGS	-	-	0.01 (0.10)	-0.18-0.20	.91	0.99
		APOE	-	-	-0.26 (0.07)	-0.40- -0.13	<.001	-22.61
		Educational attainment	-	-	0.01 (0.01)	-0.003-0.03	.11	0.99
	Interaction	PGS * Educational attainment	0.00 (-0.01-0.02)	-0.00 (-0.01-0.01)	-0.001 (0.01)	-0.01-0.01	.86	-0.10
Wealth	Main effect	PGS	-	-	0.002 (0.06)	-0.12-0.12	.98	0.20
		APOE	-	-	-0.26 (0.07)	-0.39- -0.13	<.001	-22.61
		High	-	-	-	-	-	-
		Intermediate	-	-	-0.14 (0.08)	-0.29-0.01	.08	-12.90
		Low	-	-	-0.33 (0.07)	-0.47- -0.18	<.001	-27.76
	Interaction	PGS * High	-	-	-	-	-	-
		PGS * Intermediate	-0.06 (-0.10-0.08)	-0.07 (-0.18-0.07)	-0.07 (0.07)	-0.21-0.07	.31	-7.10
		PGS * Low	0.02 (-0.1-0.14)	0.03 (-0.02-0.13)	0.03 (0.07)	-0.10-0.16	.66	2.92

Effect size is indicated by *β* coefficient and standard error (SE) from the accelerated failure time survival model; CI, confidence interval; AD, Alzheimer's disease; PGS, polygenic score.

All analyses are adjusted for age, gender, marital status, current smoking status, *APOE*-ε4 and 4 genetic principal components.

PGS, polygenic score; RERI, relative excess risk due to interaction; AP, attributable proportion; RERI=0; AP=0: no interaction or exactly equal to additivity of the individual effects of the two risk factors; RERI>0; AP>0: positive interaction or more than additivity of the individual effects of the two risk factors; RERI<0; AP<0: negative interaction or less than additivity of the individual effects of the two risk factors.

β coefficients in the AFT model were converted into time to first dementia diagnosis through the equation: $((e^{\beta}-1) \times$ mean time to dementia diagnosis).

Supplementary Table S6. Multivariate Accelerated Failure Time model estimating difference in time to the diagnosis of clinical AD dementia during the 10-year follow-up

			Additive interaction effect	
Dementia		Variables	RERI (95% CI)	AP (95% CI)
PGS score only		PGS	-	-
		APOE-ε4	-	-
Educational attainment	Main effect	PGS	-	-
		APOE-ε4	-	-
		Educational attainment	-	-
	Interaction	PGS * Educational attainment	0·01 (0·00-0·05)	0·01 (-0·002-0·05)
Wealth	Main effect	PGS	-	-
		APOE-ε4	-	-
		High	-	-
		Intermediate	-	-
		Low	-	-
	Interaction	PGS * High	-	-
		PGS * Intermediate	-0·26 (-0·27- -0·06)	-0·35 (-0·62- -0·04)
		PGS * Low	-0·23 (-0·26-0·01)	-0·29 (-0·56-0·01)

Effect size is indicated by β coefficient and standard error (SE) from the accelerated failure time survival model; CI, confidence interval; AD, Alzheimer's disease; PGS, polygenic score.

All analyses are adjusted for age, gender, marital status, current smoking status, *APOE*-ε4 and 4 genetic principal components

PGS, polygenic score; RERI, relative excess risk due to interaction; AP, attributable proportion; RERI=0; AP=0: no interaction or exactly equal to additivity of the individual effects of the two risk factors; RERI>0; AP>0: positive interaction or more than additivity of the individual effects of the two risk factors; RERI<0; AP<0: negative interaction or less than additivity of the individual effects of the two risk factors.

β coefficients in the AFT model were converted into time to first dementia diagnosis through the equation $((e^{\beta}-1)\times$ mean time to dementia diagnosis).

Supplementary Table S7. Multivariate Accelerated Failure Time model estimating difference in time to diagnosis of Alzheimer's disease during the 10-year follow-up in association with AD-$PGS_{best-fit}$ calculated using the software PRSice

			Additive interaction effect		Multiplicative interaction effect			
Dementia		Variables	RERI (95% CI)	AP (95% CI)	β (SE)	95% CI	p-value	β converted to time to clinical AD dementia a diagnosis
PGS score only		PGS	-	-	0.01 (0.05)	-0.08-0.10	.88	0.99
		APOE	-	-	-0.44 (0.12)	-0.67- -0.21	<.001	-35.15
Educational attainment	Main effect	PGS	-	-	-0.02 (0.05)	-0.12-0.07	.66	-1.96
		APOE	-	-	-0.43 (0.12)	-0.66- -0.21	<.001	-34.51
		Educational attainment	-	-	-0.15 (0.05)	-0.12- -0.07	.01	-13.76
	Interaction	PGS * Educational attainment	0.06 (-0.10-0.02)	0.07 (-0.14-0.01)	0.07 (0.04)	-0.15-0.02	.12	7.16
Wealth	Main effect	PGS	-	-	-0.06 (0.07)	-0.21-0.08	.39	-5.75
		APOE	-	-	-0.42 (0.11)	-0.63- -0.21	<.001	-33.87
		High	-	-	-	-	-	-
		Intermediate	-	-	-0.18 (0.10)	-0.39-0.02	.08	-16.27
		Low	-	-	-0.12 (0.11)	-0.34-0.11	.31	-11.17
	Interaction	PGS * High	-	-	-	-	-	-
		PGS * Intermediate	0.12 (-0.11-0.40)	0.13 (-0.13-0.27)	0.13 (0.09)	-0.04-0.31	.13	12.04
		PGS * Low	0.04 (-0.04-0.30)	0.05 (-0.08-0.20)	0.04 (0.09)	-0.13-0.22	.62	3.87

Effect size is indicated by β coefficient and standard error (SE) from the accelerated failure time survival model; CI, confidence interval; AD, Alzheimer's disease; PGS, polygenic score.

All analyses are adjusted for age, gender, marital status, current smoking status, APOE-ε4 and 4 genetic principal components.

PGS, polygenic score; RERI, relative excess risk due to interaction; AP, attributable proportion; RERI=0; AP=0: no interaction or exactly equal to additivity of the individual effects of the two risk factors; RERI>0; AP>0: positive interaction or more than additivity of the individual effects of the two risk factors; RERI<0; AP<0: negative interaction or less than additivity of the individual effects of the two risk factors.

β coefficients in the AFT model were converted into time to first dementia diagnosis through the equation: $((e^{\beta}-1) \times$ mean time to dementia diagnosis
